# Catch-up HPV vaccination status of adolescents in relation to socioeconomic factors, individual beliefs and sexual behaviour

**DOI:** 10.1371/journal.pone.0187193

**Published:** 2017-11-03

**Authors:** Maria Grandahl, Margareta Larsson, Tina Dalianis, Christina Stenhammar, Tanja Tydén, Ragnar Westerling, Tryggve Nevéus

**Affiliations:** 1 Department of Women’s and Children’s Health, Uppsala University, Uppsala, Sweden; 2 Department of Public Health and Caring Sciences, Uppsala University, Uppsala, Sweden; 3 Department of Oncology-Pathology, Karolinska Institutet, Karolinska University Hospital, Stockholm, Sweden; Rudjer Boskovic Institute, CROATIA

## Abstract

In 2012, human papillomavirus (HPV) vaccination was introduced free of charge in the Swedish national school-based vaccination programme for 10-12-year-old girls, and as catch-up vaccination for young women. In Sweden, there is an ongoing discussion about including boys in the national vaccination programme. Few studies are undertaken about adolescents’ knowledge, beliefs and HPV vaccination status in relation to socioeconomic status and sexual experience. Thus, the aim was to examine HPV catch-up vaccination status in adolescents in relation to 1) socioeconomic factors, 2) beliefs and knowledge about HPV prevention, and 3) sexual behaviour. The Health Belief Model was used as a theoretical framework. Upper secondary school students (n = 832) aged 16, randomly chosen from a larger sample, were invited to participate in conjunction with the general health interview with the school nurse. A total of 751/832 (90.3%), girls (n = 391, 52%) and boys (n = 360, 48%) completed the questionnaire. HPV vaccination was associated with ethnicity and the mothers’ education level; i.e. girls with a non-European background and girls with a less educated mother were less likely to have received the vaccine (*p*<0.01 and *p* = 0.04 respectively). Vaccinated girls perceived HPV infection as more severe (*p =* 0.01), had more insight into women’s susceptibility to the infection (*p* = 0.02), perceived more benefits of the vaccine as protection against cervical cancer (*p*<0.01) and had a higher intention to engage in HPV-preventive behaviour (*p* = 0.01). Furthermore, boys and girls were almost equally sexually experienced, although fewer girls had used condom during first intercourse with their latest partner (*p* = 0.03). Finally, HPV vaccinated girls were less likely to have unprotected sex (*p*<0.01). In summary, catch-up HPV vaccination among young girls was associated with a European background and high maternal education level, as well as more favourable beliefs towards HPV prevention and less sexual risk-taking. Further preventive measures should therefore be directed at the migrant population.

## Introduction

One decade has passed since the vaccines Gardasil and Cervarix against human papillomavirus (HPV) were introduced. These vaccines have primarily been developed to prevent infection with HPV types 16 and 18, the types responsible for roughly 70% of all cervical cancer, affecting more than 500 000 women annually [1]. They also have a preventive effect against HPV infections causing cancer in the oropharynx and the anus. Furthermore, the quadrivalent HPV vaccine Gardasil can potentially prevent a considerable number of condylomas and papillomas [2, 3].

Despite the successful impact of these vaccines worldwide, their introduction has sparked debate not only about possible side effects of the actual vaccines, but even more on assumed effects on the behaviour of the young people being vaccinated [4–6]. A fear has been voiced that the vaccines may seduce young people into becoming more sexually adventurous and lose their moral values, or even that young people with excellent morals would not need such vaccines [5, 7]. Therefore, their introduction has been challenged not only by the economic costs, but also by compromised social acceptance of the vaccines in various geographical regions or among different communities [8–10].

Previous research indicates that several factors are important for HPV vaccination status, i.e. demographic and socioeconomic factors, individual beliefs and knowledge as well as behavioural aspects. Lower socioeconomic resources correlate with lower vaccine uptake. A Danish register study showed lower HPV vaccine initiation and completion among girls with an immigrant background and girls with mothers with lower educational level [11]. A similar study conducted in Norway found lower vaccine uptake among girls with mothers in the lowest income bracket, mothers with lower educational level and mothers with an immigrant background [12]. Moreover, ethnicity and cultural norms have an impact on vaccine uptake [13–15]. Ethnicity predicted HPV vaccine uptake among adolescent girls in the catch-up cohort in the UK and among young women in Australia [13, 15]. Findings also indicate lower acceptability of HPV vaccine among ethnic minorities even after controlling for socioeconomic status [14].

Adolescents have low knowledge about HPV, especially about the link between sexual behaviour, HPV and HPV-related cancer [16–18]. According to a recent systematic review there are also gender differences, i.e. boys know less than girls [19]. The findings among adolescents in the UK indicate low knowledge and awareness about HPV vaccination [20]. The boys commonly believed that HPV only affected girls and were ill informed about the association between HPV and cancer [20]. HPV infections and HPV-related diseases have increased in the recent decades due to increased sexual risk-taking [21, 22]. Risk-taking in other domains such as alcohol and smoking has been found to correlate with risky sexual behaviour [23]. Furthermore, there are gender differences regarding beliefs about condom use, as well as actual sexual behaviour, boys being less concerned than girls [24, 25].

In 2010, HPV vaccination was offered free of charge to all girls born 1999 or later, and in 2012 the vaccine was introduced into the school-vaccination programme for all girls aged 10–12 years, and as catch-up vaccination for young women. Boys were not included in the vaccination programme at the time. These measures have had considerable impact: it is estimated that presently almost 80% of all girls receive the vaccination [26]. School nurses are responsible for all aspects of the vaccinations, including information to parents and pupils, obtaining informed consent from the former, as well as the actual administration of the vaccine. The written information given by the nurses is standardised by the government. There is an ongoing discussion about including boys in the national vaccination programme, a strategy to which the Public Health Agency of Sweden is in favour [27]. Few studies have been conducted about adolescent boys’ attitudes to HPV vaccinations: so far, most studies on males have addressed male college students or men who have sex with men [28, 29]. Consequently, it is important to examine boys’ beliefs and knowledge about HPV and HPV vaccination.

Two years ago, we conducted a cluster randomized controlled study [30], with the overall aim to improve primary prevention of HPV infection by promoting vaccination and increased condom use among upper secondary school students. This educational intervention was delivered by the school nurse in conjunction with the general health interview that was offered to all adolescents aged 16 years. At this time roughly 50–60% of all the girls had received the vaccine [20]. We found that the intervention had favourable effects on the adolescents’ beliefs towards prevention of HPV and at follow-up after three months more girls in the intervention group were vaccinated than the controls [30].

As already mentioned above, HPV vaccination status was associated with socioeconomic factors, individual beliefs, and behavioural aspects such as risk-taking and sexual experience. This study has therefore focused on the baseline data of the participants of the intervention study.

Our aims were dual:

1. To describe sexual experiences in 16-year-old adolescent girls and boys in general and for the former group also in relation to catch-up HPV vaccination status, (see item 2).
2. To investigate HPV vaccination status in female adolescents in relation to a) sociodemographic factors, b) individual beliefs and knowledge about HPV prevention, and c) sexual experiences.

It was hypothesised that vaccination against HPV was associated with a more favourable socioeconomic situation, more favourable beliefs towards HPV prevention, and less risk-taking. It was also hypothesised that there would be differences between boys and girls regarding sexual experiences and behaviour.

## Methods

### Design

This cross-sectional study is a part of the project Prevention of HPV in a school-based setting [30], with trial registration—ClinicalTrials.gov Identifier: NCT02280967. The theoretical framework was the Health Belief Model (HBM), described below. We collected the baseline data between October 2014 and February 2015. The focus of this paper is mainly on girls since only girls are offered HPV vaccination in the national vaccination programme. The study follows reports of cross-sectional studies according to STROBE (S1 File. STROBE Checklist) [31].

#### Setting

Sweden is a multicultural country of 10 million inhabitants; about 23% of the population at large, and almost 30% of those below age 18 years of age, have an immigrant background with at least one parent born abroad. The most common countries of origin outside the Nordic countries are Afghanistan, Ethiopia, Iraq, Iran, Somalia and Syria–thus, many of these families have experienced war [32, 33]. Almost all 16–19 year-olds in Sweden attend upper secondary school, which comprises theoretical and vocational programmes. During their first year all students are offered a health interview with the school nurse, focused on the individual adolescent’s health and well-being. This interview is non-compulsory but the vast majority of the students do participate [34].

#### Procedure

In 2014, school nurses working in upper secondary schools (n = 59) in Sweden were invited to participate in the project via direct contact at the national conference for school nurses. We also sent information about the study by email to the heads of the school health in the respective municipalities (n = 9). The north and the south extremities of the country were excluded for logistic reasons. In the end 20 school nurses, working in 18 schools situated in five counties (nine municipalities) in mid and western Sweden, were included. In a Supplemental file (S1 Fig. Flow chart.) as well as our earlier publication [30], more details and a flow chart regarding the recruitment process are provided. The participating schools comprised those both publicly and privately managed, offered vocationally as well as theoretically oriented education, and had a varying number of students.

In the second stage classes were randomised to be involved and thereafter adolescents in the included classes were invited to participate. The school nurse informed adolescents (n = 832) (both girls and boys) about the project orally and with a leaflet before the health interview started. Adolescents who agreed to participate gave informed written consent and completed a questionnaire individually in a separate room at the school nurse’s office. It took about 10–15 minutes to complete the questionnaire.

Eligible for inclusion were first-year upper secondary school students, aged 15 years or more, attending the general health interview with the school nurse. Students with severe cognitive learning disabilities and development disorders (e.g. students attending special schools) and students who were not able to read and write in Swedish (e.g. recently arrived immigrants) were excluded. For details of the procedure and randomisation see Grandahl et al [30]. Notably, none of the included young girls had received HPV vaccination as 10–12 year olds through the school based vaccination programme, thus all vaccinated girls had received catch-up vaccination.

#### Theoretical framework

HBM was used as a theoretical framework for this project. According to HBM, a person’s health behaviour can be explained by individual beliefs regarding health actions. HBM includes the following central constructs: *perceived susceptibility*, *perceived severity*, *perceived benefit*, *perceived barriers*, *perceived self-efficacy* and *cues to action*. In HBM, socio-demographic factors such as age, sex, ethnicity and education level as well as knowledge are recognised as modifying factors for the individual’s behaviour [35]. The theory has previously been used in behavioural research about HPV [36–40] and sexual health [41].

#### The questionnaire

The questionnaire was based on previous research [21, 30, 42, 43]. Questions about beliefs (see Table 1), knowledge and awareness about HPV and HPV vaccination (n = 24) were based on previous studies among parents [43] and school nurses [42], and comprised multiple choice alternatives and six-point verbal rating scales from”Totally agree” to”Totally disagree”, including “Do not know”. Questions about sexual health (n = 17) were based on an earlier project among university students [21] and the demographic questions (n = 14) were taken from a national questionnaire for adolescents [44]. The questionnaire has been tested for validity and reliability as described previously by Grandahl et al [30].

**Table 1:** Beliefs about HPV* and HPV vaccine–questions based on HBM**.

Central Constructs HBM	
**HBM Benefits**	The HPV vaccine is effective in preventing condyloma
	The HPV vaccine is effective in preventing cervical cancer
	I will vaccinate against HPV
**HBM Barriers**	The HPV vaccine can cause adverse effects
	It is problematic to book an appointment for HPV vaccination
	I am afraid of needles
**HBM Severity**	The HPV infection is a serious health concern
	Cervical cancer is a serious disease
**HBM Susceptibility**	Young women are at risk of contracting HPV
	Young men are at risk of contracting HPV

*HPV = Human papillomavirus

**HBM = The Health Belief Model

### Ethical considerations

We conducted the study according to the Declaration of Helsinki. All participants received oral and written information before giving their written consent. The participants were informed that participation was voluntary, that they could withdraw at any time, for any or no given reason, without incurring any negative consequences for themselves. They were also informed that only the researchers would have access to the data and that all data would be presented on a group level. Contact details to the researchers were provided in case of further questions. According to the Swedish law children above 15 years of age who understand what participation means have the right to give their own informed consent regarding participation in research. Therefore, informed consent was not obtained from the parents. We asked permission to conduct the study from both the head of the school health in each municipality and from the heads of the individual schools. Approval was acquired from The Regional Ethical Review Board of Uppsala University (D.nr.2013/324).

### Statistical analysis

Data were analysed using SPSS for IBM (version 20; IBM Corp., Armonk, NY, USA) (S2 File. Data set). We defined sexual risk-taking as unprotected intercourse either a) at sexual debut, b) at the first occasion with the latest partner or c) during latest sexual activity. Moreover, the respondent was defined as having sexual experience if she or he had ever had sexual intercourse or participated in oral sex. Intention to prevent HPV related cancer was defined as agreeing or partly agreeing to the statements: *“I intend to use a condom if I have sex with a new partner”* and *“I intend to attend Pap-smear testing”*. We created a knowledge score by computing the responses to ten of the statements about HPV and the HPV vaccine. Each of the statements could yield a maximum of four points if the respondent totally agreed with a correct or totally disagreed with an incorrect statement. The response alternatives “*neither agree nor disagree”* and “*do not know”* were given zero points. The total knowledge score could thus add up to a maximum of 40 points. We investigated differences between groups (girls/boys, vaccinated/non-vaccinated, sexual risk-takers/non risk-takers) with Chi-square test and Fisher’s exact text for nominal-scaled variables, with Mann Whitney *U*-test for ordinal-scaled variables and with Student’s t-test for continuous variables. In order to make comparisons, we collapsed some of the variables into dichotomous variables. Country of origin was thus dichotomised into European and non-European; parents’ education into low (up to 12 years of schooling) and high; parents’ occupation into either working/studying or neither; smoking and snuff use into either and none; and alcohol binge drinking into never and sometimes. We used a multivariate logistic regression model to investigate factors associated with HPV vaccination status among the girls. Sociodemographic variables such as parents’ country of origin, education, and occupation correlated highly, as did the HPV-related variables. We therefore chose to enter only two non-correlating sociodemographic variables; country of origin and mother’s education, the calculated knowledge score and the variable “discussed HPV with other than parents” in the multivariate regression model. A p-value of *p*<0.05 was considered statistically significant. The missing values were few and did not exceed 3% for any of the items.

## Results

A total of 751/832 (90.3%), 391 girls and 360 boys, agreed to participate. Table 2 presents the characteristics of the participants and their parents. As indicated in Table 2, about one quarter of the participants had an immigrant background. The average age of both girls and boys was 16 years, and there were no differences in parental characteristics. Girls tended to smoke more often than boys, whereas boys used more snuff than girls did. There were no differences between the sexes with regard to alcohol consumption.

**Table 2:** Characteristics of the participants and their parents.

Variable	Girls n = 391 n (%)	Boys n = 360 n (%)	p-value
**Country of birth**			0.60
Sweden	350 (89.5)	330 (91.9)	
Another Nordic country	2 (0.5)	2 (0.5)	
Europe	6 (1.5)	6 (1.7)	
Outside Europe	33 (8.4)	21(5.8)	
**Mother’s country of birth**			0.33
Sweden	301 (77.0)	282 (79.3)	
Another Nordic country	13 (3.3)	15 (4.2)	
Europe	23 (5.9)	10 (2.8)	
Outside Europe	51 (13.0)	46 (12.8)	
**Father’s country of birth**			0.36
Sweden	294 (75.6)	284 (78.9)	
Another Nordic country	12 (3.1)	13 (3.6)	
Europe	27 (6.9)	13 (3.6)	
Outside Europe	51 (13.0)	47 (13.1)	
**Study programme**			0.13
Theoretical (university preparation)	153 (39.8)	154 (45.6)	
Vocational	231 (60.2)	184 (54.4)	
**Mother’s education**			0.54
9 years	20 (5.1)	17 (4.7)	
12 years	122 (31.4)	116 (32.3)	
More than 12 years	180 (46.3)	151 (42.1)	
**Father’s education**			0.86
9 years	29 (7.5)	30 (8.3)	
12 years	149 (38.3)	141 (39.2)	
More than 12 years	109 (28.0)	104 (28.9)	
**Mother’s occupation**			0.45
Working	327 (84.3)	309 (86.1)	
Studying	9 (2.3)	10 (2.8)	
Parental leave	6 (1.5)	1 (0.3)	
Sick leave	22 (5.7)	23 (6.4)	
Unemployed	10 (2.6)	6 (1.7)	
Other	14 (3.6)	10 (2.8)	
**Father’s occupation**			0.92
Working	347 (89.0)	316 (88.5)	
Studying	3 (0.8)	2 (0.6)	
Parental leave	11 (2.8)	13 (3.6)	
Sick leave	10 (2.6)	10 (2.8)	
Unemployed	19 (4.9)	16 (4.5)	
**Smoking**			<0.001
No	298 (76.2)	318 (88.3)	
Yes, sometimes	44 (11.3)	24 (6.7)	
Yes, daily	49 (12.5)	18 (5.0)	
**Snuff use (oral tobacco)**			<0.001
No	379 (96.9)	309 (85.8)	
Yes, sometimes	11 (2.8)	22 (6.1)	
Yes, daily	1 (0.3)	29 (8.1)	
**Alcohol binge drinking**			0.27
Never	239 (61.3)	242 (67.2)	
Seldom	81 (20.8)	62 (17.2)	
Once/month	61 (15.6)	45 (12.5)	
Once/week	9 (2.3)	11 (3.1)	

Missing values excluded from the analysis

### Sexual experiences and HPV vaccination status

Around half of the respondents had had sexual intercourse as shown in Table 3. Boys and girls were almost equally experienced in this respect. The reported ranges in sexual debut were mainly between 12 and 17 years of age, while two students reported 11 years of age and one student reported 19 years of age. Girls reported slightly older partners than boys did, had given oral sex to a higher extent, were more likely to have been tested for a sexually transmitted infection (STI), and were more often vaccinated against HPV.

**Table 3:** Sexual experiences, HPV* vaccination status and contraceptive use among sexually active participants.

Variable	Girls	Boys	p-value
Had had intercourse	198 (51.0)	167 (47.3)	ns
Age at first intercourse (mean)	14.6	14.8	ns
Age of partner at first intercourse (mean)	16.1	15.2	<0.001
Age of partner at latest intercourse (mean)	18.0	16.2	<0.001
Number of life-time sexual partners (mean)	3.4	3.4	ns
Number of sexual partners latest 3 months (mean)	1.0	1.0	ns
New sexual partner latest 3 months (n = 202/170)	52 (25.7)	47 (27.6)	ns
Given oral sex	149 (38.4)	96 (27.9)	0.01
Received oral sex	147 (38.2)	118 (34.2)	ns
Age at first oral sex (mean)	15.0	15.0	ns
Used condom at latest oral sex	17 (7.9)	10 (4.9)	<0.001
Tested for STI**	82 (21.2)	34 (9.6)	<0.001
Ever had an STI	8 (2.1)	5 (1.4)	ns
Vaccinated against HPV	216 (55.5)	1 (0.3)	<0.001
**Contraception during first intercourse (n = 198/167)**			
None	38 (19.0)	23 (13.9)	ns
Condom	143 (72.2)	118 (70.7)	ns
Withdrawal	9 (4.5)	8 (4.8)	ns
Oral contraceptive pill	34 (17.1)	31 (18.8)	ns
Other	3 (1.5)	5 (3.0)	ns
**Contraception during first intercourse with latest partner (n = 187/158)**			
None	40 (21.3)	25 (15.8)	ns
Condom	79 (42.2)	94 (59.5)	0.03
Withdrawal	5 (2.7)	7 (4.4)	ns
Oral contraceptive pill	72 (38.5)	49 (31.0)	ns
Other	16 (8.6)	4 (2.5)	ns
**Contraception during latest intercourse (n = 193/158)**			
None	41 (21.1)	27 (17.1)	ns
Condom	57 (29.5)	81 (51.3)	0.02
Withdrawal	5 (2.6)	5 (3.2)	ns
Oral contraceptive pill	90 (46.6)	52(32.9)	0.01
Other	18 (9.1)	9 (5.7)	ns

Missing values excluded from the analysis

*HPV = Human papillomavirus

** STI = Sexually transmitted infection

### Beliefs and knowledge about HPV among vaccinated and non-vaccinated girls

Table 4 shows differences between vaccinated and non-vaccinated girls with regard to the HBM-related beliefs, although no differences were found in knowledge about HPV. Vaccinated girls perceived HPV infection as more severe, had more insight into women’s susceptibility of the infection, perceived more benefits about the vaccines protective effect against cervical cancer and had a higher intention to engage in HPV-preventive behaviour. Non-vaccinated girls were more ignorant about how to make an appointment for HPV-vaccination.

**Table 4:** Beliefs and knowledge about HPV* and HPV vaccination with respect to vaccination status.

HBM**-related construct	Statements	Vaccinated Agree/ do not agree/do not know n = 216	Not vaccinated Agree/ do not agree/do not know n = 175	p-value (MannWhitney *U-*test)
		(%)	(%)	
Severity	HPV can lead to a serious disease	68/5/26	59/8/33	0.02
	Cervical cancer can be life-threatening	63/15/22	57/13/31	0.01
Susceptibility	Women can be infected with HPV	73/7/21	65/6/30	0.02
	Men can be infected with HPV	10/46/44	12/35/53	0.33
Knowledge	HPV can spread through sexual contact	48/17/36	47/14/40	0.41
	You always notice if you are HPV infected	4/44/52	5/40/55	0.65
	HPV can cause other types of cancer	13/14/74	17/17/66	0.12
Barrier	The HPV vaccine has severe side effects	17/45/38	23/25/52	0.56
	It’s difficult to make an appointment for HPV vaccination	2/63/35	2/39/58	<0.01
	I am afraid of needles	38/61/1	45/51/5	0.08
Benefits	HPV vaccination protects against condyloma	12/27/61	18/18/63	0.75
	HPV vaccination protects against cervical cancer	73/2/23	57/8/35	<0.01
Intention to behaviour	I intend to use condom if I have sex with a new partner	86/8/7	78/8/14	0.01
	I intend to attend Pap-smear testing (only girls)	54/11/36	40/15/46	0.01

Missing values excluded from the analysis

*HPV = Human papillomavirus

**HBM = The Health Belief Model

There were no differences in separate knowledge items about HPV and HPV vaccination among vaccinated or non-vaccinated girls. However, when computed together to form a knowledge score, vaccinated girls scored higher (Table 4).

Differences between HPV vaccinated and non-vaccinated girls with respect to background variables, sexual experience and other HPV related variables were also examined. The results of the bivariate analyses are shown in Table 5. There were several differences between the groups with respect to their background variables, but no differences in sexual experience. All HPV related variables differed between vaccinated and non-vaccinated girls (Table 5).

**Table 5:** Bivariate analysis of differences between HPV* vaccinated and non-vaccinated girls.

Variable	Vaccinated % (n = 216)	Non vaccinated % (n = 175)	p-value
Non-European background	7	27	<0.01
Theoretical study programme	43	35	ns
Mother’s education more than 12 years	62	48	0.02
Father’s education more than 12 years	44	30	0.03
Mother working/studying	90	82	0.02
Father working/studying	95	87	<0.01
Smoking	18	31	<0.01
Snuff use (Oral tobacco)	2	4	ns
Alcohol binge drinking	39	38	ns
Had had sexual intercourse	50	52	ns
Given oral sex	37	40	ns
Received oral sex	39	37	ns
Tested for STI**	22	21	ns
Ever had an STI	2	2	ns
Heard about HPV	38	27	0.02
Heard about HPV vaccine	44	31	0.02
Heard about vaccine against cervical cancer (yes/no)	99	92	<0.01
Discussed HPV with parents	78	60	<0.01
Discussed HPV with other	58	38	<0.01
Friend is vaccinated against HPV	90	67	<0.01
Knowledge index (mean based on 10 statements)	18.7	16.0	<0.01

*HPV = Human papillomavirus

**STI = Sexually transmitted infection

A multivariate logistic regression model including selected variables differing significantly in the bivariate analysis was performed as described in the Methods section. The result is displayed in Table 6, showing that non-European background and maternal education level remained associated with HPV vaccination.

**Table 6:** Multivariate logistic regression models of variables associated with HPV* vaccination among girls.

Variables	OR	95% CI	p-value
Non-European background (no/yes. Reference European background)	0.22	0.07–0.70	0.01
Mother’s education (low/high. Reference low)	2.96	1.07–8.21	0.04
Discussed HPV with other (yes/no. Reference yes)	0.56	0.34–0.91	0.19
Knowledge index (mean based on 10 statements)**	0.98	0.95–1.00	0.06

*HPV = Human papillomavirus

**(continues variable; between 0 and 40 points based on 10 statements)

Finally, we examined sexual risk-taking in relation to HPV vaccination status and found that vaccinated girls had less unprotected first intercourse with their latest partner (12.7% vs. 31.8%, *p<*0.01), and less unprotected latest intercourse (13.9% vs. 30.6%, *p* = 0.01) compared to their non-vaccinated peers. No difference was found in relation to first intercourse (16.5% vs. 22.2%). The girls’ intention to prevent HPV infection in the future did not differ with respect to demographic variables, such as country of birth or parental factors.

## Discussion

This study aimed to examine catch-up HPV vaccination status in youth in relation to socioeconomic factors, beliefs and knowledge about prevention of HPV, and sexual behaviour among boys and girls. The study showed that there were differences in beliefs and in health behaviour regarding sexual risk-taking. Differences in beliefs were that vaccinated girls perceived HPV infection as more severe, had more insight into women’s susceptibility of the infection, perceived more benefits about the vaccines protective effect against cervical cancer and had a higher intention to engage in HPV-preventive behaviour. Differences in sexual risk-taking were that HPV vaccinated girls had less unprotected first intercourse with their latest partner, and less unprotected latest intercourse compared to their non-vaccinated peers. However, the girls’ intention to prevent HPV infection in the future did not differ significantly with respect to demographic variables, such as country of birth or parental factors.

Our hypothesis, that vaccination against HPV was associated with a more favourable socioeconomic status, more favourable beliefs towards HPV prevention, and less risk-taking was partly confirmed. In the bivariate analysis differences were found between vaccinated and non-vaccinated girls depending on parental educational level and parental working status, discussions of HPV vaccine with parents and others and if a friend was vaccinated against HPV. However, in the final multivariate logistic regression model the only variables associated with HPV vaccination were country of birth and mother’s level of education.

The difference in HPV vaccination status between ethnic groups was not surprising since the most common origins of immigrants in Sweden today are from non-European countries such as Afghanistan, Syria, Ethiopia, and Somalia. Girls with an immigrant background are vulnerable groups that often need catch-up vaccinations [45]. Cultural norms and religious beliefs can have an impact on health behaviour and HPV vaccination status [13, 14]. Bowyer et al found ethnicity independently associated with vaccine uptake, and adolescent girls with an immigrant background had lower vaccine uptake [13]. Similar findings have been found in registry studies undertaken in Norway and Denmark [11, 12]. More specifically, differences were found in vaccine initiation and completion, depending on socioeconomic factors such as parental education, employment and income level, with lower vaccination status among girls with immigrant or poorly educated mothers [11, 12]. However, national vaccination programmes can reduce disparities in vaccine uptake due to socioeconomic factors, a fact which emphasises the need for school-based vaccinations.

Generally, the responses of the HPV vaccinated girls revealed more informed beliefs and sound intentions than those of their non-vaccinated peers. This is not surprising, since individual beliefs about HPV vaccination surely influence the decision to take the vaccine. It should also be noted that parents have to consent to vaccination for children and parental beliefs are thus important as well [4, 43, 46–49]. This fits well with our finding in the bivariate analyses that HPV vaccination was associated both with discussions with parents or others and the presence of vaccinated friends. Peers evidently have a substantial influence on the adolescents’ decisions, an observation which is supported by HBM. The discussions with friends might have strengthened the girls’ self-efficacy and influenced the girls to vaccinate, i.e. they might have functioned as cues to action.

According to HBM, knowledge is a modifying factor for the individual health behaviour, yet, in the final regression model the knowledge index did not remain associated with HPV vaccination. However, the knowledge about HPV and HPV vaccine was low among the participants overall. This lack of knowledge was disheartening, but has been described previously [17, 30, 43, 50]. There is surely a need for more public education in this important field, especially about the link between HPV infection and cancer [17, 30]. As found in previous studies educational intervention can have favourable effects on adolescents’ awareness and beliefs [18, 30, 50] and also on vaccination status [30].

For girls whose mothers had a low education level it was less common to be vaccinated. For girls born outside Europe, however, the lower odds ratios for being vaccinated persisted also after taking the level of knowledge among the girls into account. A previous study has shown positive attitudes towards preventive measures for cervical cancer among migrant women in Sweden [51]. However, the health literacy, i.e. the ability to access, understand value and use health information, is often low among migrant groups in Sweden. In order to not only receive information about vaccination, but also to value and use the information and take action to vaccinate, more advanced health literacy is needed. The lower vaccination rates among non-European girls thus may be due to lower levels of complex health literacy as well as to poor knowledge about the Swedish healthcare system. Preventive strategies may need to take this into account by not only informing about vaccinations, but also give room for an active dialogue about how to value and use this information [52].

Boys and girls were almost equally sexually experienced, although girls reported slightly older partners than boys did, had given oral sex to a higher extent, and were more likely to have been tested for an STI. This is not surprising, since the vast majority attending youth clinics in Sweden are girls. Previous studies also indicate that boys have lower knowledge about HPV and the HPV vaccine and are less worried about sexual health risks [19, 20, 23–25]. Thus, it is important to provide information to both boys and girls, especially about the link between sexual behaviour, HPV infection and HPV-related cancer. Sadly, the low condom use is a common finding in different contexts among youth, which might indicate the need for gender-neural vaccinations, i.e., HPV vaccination to both boys and girls.

No differences were found in experience of intercourse and oral sex among HPV vaccinated and non-vaccinated girls. HPV vaccination was not associated with earlier sexual debut or more sexual risk-taking (unprotected sexual intercourse). This very important finding needs to be emphasized, since the fear that HPV vaccination could promote “promiscuity” is a recognized obstacle against vaccination [4, 5, 7]. Our data show that this fear is ungrounded. Our findings were also in line with previous research undertaken in the USA among girls and young women aged 13–24 [7]. If authorities and the community at large acted in accordance with this knowledge more adolescents would become vaccinated and, in the end, lives would be saved.

### Strengths and weaknesses

The questionnaire was validated and a high response rate of 90.3% was obtained [30]. The strength of a school-based project is that it reaches all adolescents regardless of sex, socio-economic status, ethnicity or cultural background. The included adolescents represented a wide range of schools situated in different socioeconomic and geographic areas. The participants attended both theoretical and vocational programmes. The percentage of adolescents with an immigrant background, sexually experienced adolescents as well as the number of HPV vaccinated girls, were representative for the Swedish population in general. Taken together, this suggests that the present findings can, with a fair degree of certainty, be generalized to the population at large. The target group, adolescents aged 16 years, is adequate since this is a time in life when many become sexually active.

The participants completed the questionnaire in a separate room at the school nurse’s office. The questionnaires were properly completed and no extraordinary outliers were found in the data. As in all studies using self-reported questionnaires, there is a risk of participant over- or under-reporting and recall bias, although we consider this risk small in the present study. Furthermore, this survey had a cross-sectional design, which did not allow for conclusions regarding causes and effects. Finally, we did not know exactly when the included adolescents received the vaccination in relation to their sexual experiences.

### Clinical implication

In Sweden, HPV vaccination is included in the national vaccination programme offered free of charge by school-nurses in the school health services. The lower degree of HPV catch-up vaccination among immigrant girls needs to be emphasized since in Sweden, many girls come from low-income countries with poor vaccine coverage. In order to bridge the gap and enhance informed consents based on actual facts rather than emotional, cultural, or religious beliefs this study suggests that we can recommend that parents and adolescents are provided with the evidence-based fact that HPV vaccination neither contributes to earlier sex debut nor to more risk-taking sexual behaviour. Since the school health and especially the school nurses are key persons for successful HPV vaccination programmes this information could be distributed by them. Since the knowledge about HPV and HPV vaccine still is low we also recommend that pupils, both boys and girls, are informed about the virus and the vaccine not only at time for the vaccination, but continuously during their school period. This is in line with the national goal of equal health for the entire population. Uniquely, the school health services reach all adolescents regardless of socioeconomic status or country of origin.

### Conclusions

Catch-up HPV vaccination among young girls was associated with a European background, a high maternal education level, more favourable beliefs towards HPV prevention, and less sexual risk-taking. Further preventive measures should therefore be directed at migrant adolescent girls and boys and their parents.

## Supporting information

S1 File STROBE checklist. (DOCX)

S1 Fig Flow chart. (JPEG)

S2 File Data set. (SAV)
