# Supplementary material for: Catch-up HPV vaccination status of adolescents in relation to socioeconomic factors, individual beliefs and sexual behaviour
Source: PLoS One. 2017 Nov 3;12(11):e0187193. doi: 10.1371/journal.pone.0187193 (PMC5669438; doi:10.1371/journal.pone.0187193)
Supplement: S1 File — (DOCX) [file pone.0187193.s001.docx]

**STROBE 2007 (v4) Statement—Checklist of items that should be included in reports of *cross-sectional studies***

| **Section/Topic** | Item # | Recommendation | Reported on page # |
| --- | --- | --- | --- |
| **Title and abstract** | 1 | (*a*) Indicate the study’s design with a commonly used term in the title or the abstract | Page 2 |
|  |  | (*b*) Provide in the abstract an informative and balanced summary of what was done and what was found | Page 2 |
| Introduction | | |  |
| Background/rationale | 2 | Explain the scientific background and rationale for the investigation being reported | Pages 3-5 |
| Objectives | 3 | State specific objectives, including any prespecified hypotheses | Pages 5-6 |
| Methods | | |  |
| Study design | 4 | Present key elements of study design early in the paper | Page 6 |
| Setting | 5 | Describe the setting, locations, and relevant dates, including periods of recruitment, exposure, follow-up, and data collection | Pages 6-8, Supplemental file S1. Figure 1. |
| Participants | 6 | (*a*) Give the eligibility criteria, and the sources and methods of selection of participants | Page 7-8 |
| Variables | 7 | Clearly define all outcomes, exposures, predictors, potential confounders, and effect modifiers. Give diagnostic criteria, if applicable | Page 8 |
| Data sources/ measurement | 8* | For each variable of interest, give sources of data and details of methods of assessment (measurement). Describe comparability of assessment methods if there is more than one group | Page 8-11 |
| Bias | 9 | Describe any efforts to address potential sources of bias | Pages 6-8, and Strengths and weaknesses page 24-25 |
| Study size | 10 | Explain how the study size was arrived at | Page 5-7. This paper presents the baseline data among the adolescents’. For more information (power analysis etc) see Grandahl et al, 2016 |
| Quantitative variables | 11 | Explain how quantitative variables were handled in the analyses. If applicable, describe which groupings were chosen and why | Pages 10-11 |
| Statistical methods | 12 | (*a*) Describe all statistical methods, including those used to control for confounding | Pages 10-11 |
|  |  | (*b*) Describe any methods used to examine subgroups and interactions | N/A |
|  |  | (*c*) Explain how missing data were addressed | Page 11. The adolescents’ had completed the questionnaires thoroughly (they completed the questionnaire individually at the school nurses´ office). The missing values were few and did not exceed 3% for any of the items.  The external validity is discussed in Strengths and weaknesses on page 20. |
|  |  | (*d*) If applicable, describe analytical methods taking account of sampling strategy | N/A |
|  |  | (*e*) Describe any sensitivity analyses | N/A |
| **Results** |  |  |  |
| Participants | 13* | (a) Report numbers of individuals at each stage of study—eg numbers potentially eligible, examined for eligibility, confirmed eligible, included in the study, completing follow-up, and analysed | As mentioned earlier, this is discussed in Strengths and Limitations on pages 24-25. The Flow chart is presented in Supplemental file. S2. Figure 1. |
|  |  | (b) Give reasons for non-participation at each stage | N/A |
|  |  | (c) Consider use of a flow diagram | N/A |
| Descriptive data | 14* | (a) Give characteristics of study participants (eg demographic, clinical, social) and information on exposures and potential confounders | Table 2 and Strengths and weaknesses page |
|  |  | (b) Indicate number of participants with missing data for each variable of interest | As mentioned earlier and described in Methods, the missing values were few and did not exceed 3% for any of the items. |
| Outcome data | 15* | Report numbers of outcome events or summary measures | See tables 1-6 and Result pages 11-18 |
| Main results | 16 | (*a*) Give unadjusted estimates and, if applicable, confounder-adjusted estimates and their precision (eg, 95% confidence interval). Make clear which confounders were adjusted for and why they were included | Results pages 11-18, Tables 3-6 |
|  |  | (*b*) Report category boundaries when continuous variables were categorized | Results pages 11-18 |
|  |  | (*c*) If relevant, consider translating estimates of relative risk into absolute risk for a meaningful time period | N/A |
| Other analyses | 17 | Report other analyses done—eg analyses of subgroups and interactions, and sensitivity analyses | N/A |
| Discussion |  |  |  |
| Key results | 18 | Summarise key results with reference to study objectives | Page 18 |
| Limitations | 19 | Discuss limitations of the study, taking into account sources of potential bias or imprecision. Discuss both direction and magnitude of any potential bias | Page 21-22 |
| Interpretation | 20 | Give a cautious overall interpretation of results considering objectives, limitations, multiplicity of analyses, results from similar studies, and other relevant evidence | Pages 18-23 |
| Generalisability | 21 | Discuss the generalisability (external validity) of the study results | Page 21-22 |
| Other information |  |  |  |
| Funding | 22 | Give the source of funding and the role of the funders for the present study and, if applicable, for the original study on which the present article is based | This work was supported by [the Swedish Cancer Society] grant number [130744], [Uppsala-Örebro Regional Research Council] grant number [RFR-387561/476021]. The sponsors had no role in the design of the study or in how it was conducted, including collection, management, analysis, and interpretation of the data as well as preparation, review, or approval of the manuscript and finally, the decision to submit the manuscript for publication. |

*Give information separately for cases and controls in case-control studies and, if applicable, for exposed and unexposed groups in cohort and cross-sectional studies.

**Note:** An Explanation and Elaboration article discusses each checklist item and gives methodological background and published examples of transparent reporting. The STROBE checklist is best used in conjunction with this article (freely available on the Web sites of PLoS Medicine at http://www.plosmedicine.org/, Annals of Internal Medicine at http://www.annals.org/, and Epidemiology at http://www.epidem.com/). Information on the STROBE Initiative is available at www.strobe-statement.org.
