# Supplementary figures and images for: Catch-up HPV vaccination status of adolescents in relation to socioeconomic factors, individual beliefs and sexual behaviour
Source: PLoS One. 2017 Nov 3;12(11):e0187193. doi: 10.1371/journal.pone.0187193 (PMC5669438; doi:10.1371/journal.pone.0187193)

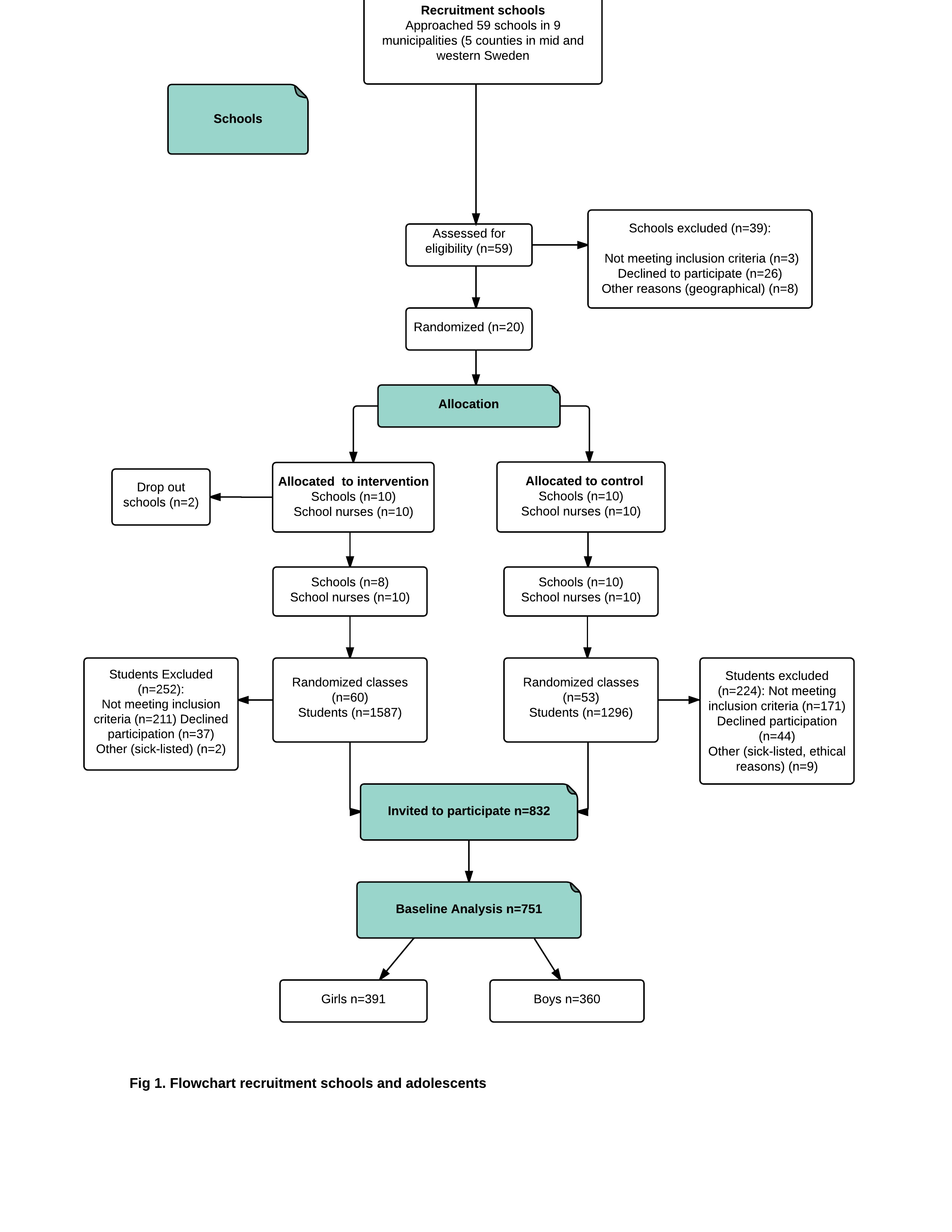

Supplement: S1 Fig — (JPEG) [file pone.0187193.s002.jpeg]
